# Geographic variation in spatial accessibility of U.S. healthcare providers

**DOI:** 10.1371/journal.pone.0215016

**Published:** 2019-04-09

**Authors:** Keith B. Naylor, Joshua Tootoo, Olga Yakusheva, Scott A. Shipman, Julie P. W. Bynum, Matthew A. Davis

**Affiliations:** 1 University of Michigan Medical School, Department of Internal Medicine, Division of Gastroenterology and Hepatology, Ann Arbor, Michigan, United States of America; 2 Institute for Healthcare Policy and Innovation, University of Michigan, Ann Arbor, Michigan, United States of America; 3 National Center for Geospatial Medicine, Rice University, Houston, Texas, United States of America; 4 University of Michigan School of Nursing, Ann Arbor, Michigan, United States of America; 5 University of Michigan School of Public Health, Ann Arbor, Michigan, United States of America; 6 The Dartmouth Institute for Health Policy and Clinical Practice, Geisel School of Medicine at Dartmouth, Hanover, New Hampshire, United States of America; 7 University of Michigan Medical School, Department of Internal Medicine, Geriatric and Palliative Medicine, Ann Arbor, Michigan, United States of America; 8 University of Michigan Institute for Social Research, Ann Arbor, Michigan, United States of America; The University of the South Pacific, FIJI

## Abstract

**Background:**

Growing physician maldistribution and population demographic shifts have contributed to large geographic variation in healthcare access and the emergence of advanced practice providers as contributors to the healthcare workforce. Current estimates of geographic accessibility of physicians and advanced practice providers rely on outdated “provider per capita” estimates that have shortcomings.

**Purpose:**

To apply state of the art methods to estimate spatial accessibility of physician and non-physician clinician groups and to examine factors associated with higher accessibility.

**Methods:**

We used a combination of provider location, medical claims, and U.S. Census data to perform a national study of health provider accessibility. The National Plan and Provider Enumeration System was used along with Medicare claims to identify providers actively caring for patients in 2014 including: primary care physicians (i.e., internal medicine and family medicine), specialists, nurse practitioners, and chiropractors. For each U.S. ZIP code tabulation area, we estimated provider accessibility using the Variable-distance Enhanced 2 step Floating Catchment Area method and performed a Getis-Ord Gi* analysis for each provider group. Generalized linear models were used to examine associations between population characteristics and provider accessibility.

**Results:**

National spatial patterns of the provider groups differed considerably. Accessibility of internal medicine most resembled specialists with high accessibility in urban locales, whereas relative higher accessibility of family medicine physicians was concentrated in the upper Midwest. In our adjusted analyses independent factors associated with higher accessibility were very similar between internal medicine physicians and specialists–presence of a medical school in the county was associated with approximately 70% higher accessibility and higher accessibility was associated with urban locales. Nurse practitioners were similar to family medicine physicians with both having higher accessibility in rural locales.

**Conclusions:**

The Variable-distance Enhanced 2 step Floating Catchment Area method is a viable approach to measure spatial accessibility at the national scale.

## Introduction

By the year 2020, the Health Resources and Services Administration (HRSA) estimates that there will be a shortage of 20,400 primary care physicians in the United States.[1] Policymakers have long debated potential solutions to the national shortfall of physicians–of which, the most straightforward being to simply increase supply.[2] However, approaches aimed at increasing the number of physicians graduating from medical schools neglect to consider the financial incentive for students to enter into procedural and surgical based non-primary care specialties and the forces that drive physicians to practice in already oversupplied locales. Interestingly, in part due to strategic efforts coupled with changes in professional education, advanced practice providers such as nurse practitioners (NPs) and physician assistants have played an increasingly important role in the U.S. healthcare workforce.[3, 4] In 2010, greater than 55,000 NPs were practicing primary care in the U.S., and the American Association of Nurse Practitioners estimates that of the greater than 23,000 NPs who graduated in 2015–2016, 85% were trained in primary care.[5, 6]

Although sobering, national healthcare workforce projections, particularly for primary care, mask substantial geographic variation. The healthcare workforce is not distributed equally across the United States–while some areas struggle to provide basic healthcare services others have an abundance of healthcare providers.[7] Such geographic imbalance in the healthcare workforce is not unique to the U.S.[8–10] and a common observation is the high concentration of providers within urban and/or affluent areas, versus a relative undersupply in rural and/or low-income areas. This has been clearly demonstrated in the distribution of U.S. surgical services.[11] Likewise, the number of primary care physicians also increases with greater urbanization, from 39.8 per 100,000 residents in non-metropolitan areas to 53.3 in large central metropolitan areas.[12] Financial incentives, recruitment, career development opportunities, infrastructure and staffing, workload and autonomy, and professional work environment have all been shown to affect where physicians practice.[13] Yet, very little is known about the factors that influence where non-physicians such as nurse practitioners practice, nor the extent to which the workforce varies geographically.

To date, previous studies that have examined geographic variation in the physician workforce have been limited to large area units (e.g., counties, hospital service regions, and primary care service areas) and rely on “per capita” measures expressed as a provider per population ratios, reflecting the availability of providers.[14, 15] Such measures have significant shortcomings in that they assume patients within regions have equal access and imply that administrative boundaries are impermeable (i.e., patients seek care only within the assigned regions).[16–18] Clearly, these are not realistic assumptions and the lack of accounting for the distance to services and boundary crossing affects the accuracy of the estimates for provider accessibility. The Variable distance Enhanced 2 step Floating Catchment Area (VE2SFCA) method is an approach to measuring spatial accessibility that was first developed by Luo and Wang and modified by others.[16–18] The VE2SFCA method accounts for provider availability, border crossing, and the effects for distance decay of utilization (i.e., adjusts for proximity to services).[19] However, the VE2SFCA has only been applied to smaller geographic areas.[12, 17, 20, 21] Accurate estimates of provider spatial accessibility on the national scale would be extremely valuable to policymakers.

Therefore, we conducted a national study to compare spatial accessibility of key physician and non-physician groups using state of the art geospatial methods. Specifically, our objectives were twofold: (1) to describe spatial accessibility of physician and non-physician clinician groups using the VE2SFCA method and (2) to examine population factors associated with higher spatial accessibility. We specifically examine spatial accessibility of medical providers including primary care physicians (internal medicine and family medicine, separately), specialists, and nurse practitioners. As an example for a non-physician group that operates completely outside traditional medicine we also examine spatial accessibility of U.S. chiropractors.

## Methods

We used a combination of national data on provider location and administrative claims to estimate spatial accessibility of primary care physicians (comparing internal medicine to family medicine), specialists, nurse practitioners, and chiropractors using the VE2SFCA method.[16–18] We then merged data from the U.S. Census Bureau to examine population factors associated with spatial accessibility.

### Identification of providers

The 2014 National Plan and Provider Enumeration System (NPPES) was used to identify healthcare providers including: family medicine physicians, internal medicine physicians, specialists, nurse practitioners, and chiropractors.[22] Provider types were classified based on the specialty codes reported in the NPPES, Table 1. Specialists included both surgical and medical specialties and subspecialties.

**Table 1:** Specialty codes used to identify providers, number of active U.S. providers, and mean provider to population ratios.

	NPPES specialty code(s)	No. of active providers	Mean provider to population ratio (SD)
Family medicine physicians	08	90,870	0.31 (0.28)
Internal medicine physicians	11	178,660	0.38 (0.54
Specialists	02, 03, 04, 05, 06, 07, 09, 10, 13, 14, 16, 17, 18, 20, 22, 23, 24, 25, 26, 27, 28, 29, 30, 33, 34, 36, 39, 40, 44, 46, 66, 72, 76, 77, 78, 79, 81, 83, 84, 85, 86, 90, 91, 92, 93, 94, 98	391,621	0.82 (1.22)
Nurse practitioners	50	79,790	0.24 (0.26)
Chiropractors	35	44,040	0.14 (0.14)

Abbreviations: NPPES, National Plan and Provider Enumeration System; SD, standard deviation

In order to identify providers who were actively caring for patients, we linked all NPPES data to the 2014 Medicare Part B 20% sample claims file. Analyses were restricted to active providers based on the presence of one or more claims within the 20% Medicare Carrier file. Provider practice location was identified from the addresses in the NPPES and geocoded to transform the physical addresses to point feature data.

### Estimation of provider spatial accessibility

We estimated provider spatial accessibility using the VE2SFCA method. This approach has distinct advantages over simple per capita estimates including: a decreased reliance on administrative boundaries; allowance for cross-border interactions; and an approximation for the effects for distance decay of utilization behavior. For the purposes of the study, spatial accessibility is based upon the concept of “potential spatial access”, described by Kahn as the availability of a service moderated by space, or the distance variable.[23] Our measure was constructed based on both geocoded 2014 NPPES and 2010 US Census block level population data aggregated to the ZCTA level.

To calculate a drive time-based service area for each practice location, we used an origin destination matrix describing the network based distance and time relationships with the health provider geocoded point location as the origin and the population weighted centroids for each ZCTA as the destination. Population weighted centroids were calculated for ZCTAs using nested 2010 US Census Block population data. ZCTA centroids were assigned the closest road segment for origin destination analysis. The origin destination matrix provided network based drive time and distance relationships for all geocoded practice locations and all ZCTA centroids within a 60-minute drive time threshold. Distance and drive time measures were calculated for automobiles using public roads exclusively. Speed limits and traffic restrictions were applied. Local traffic conditions such as day of the week and time of day were not considered in the analysis.

For each geocoded point practice location, we aggregated all ZCTA populations whose centroids fell within an initial travel time (t_0_) of 15 minutes. If the summed aggregate population value was less than 3,500 individuals, the initial travel time (t_0_) was increased in 15-minute increments (t_0+15 minutes_) until the aggregated population reached the 3,500 threshold. For primary medical care, ratios below one provider per 3,500 persons (1:3,500) are considered health professional shortage areas as defined by the United States Department of Health and Human Services.[16, 24, 25] The travel time at which the 1:3,500 threshold was satisfied was used to define the catchment area for the geocoded practice location of interest.

To account for the decreasing likelihood that individuals utilize a resource as distance to the resource increases, we adapted distance decay weights W_ij_ directly from Luo and Whippo, where t_ij_ represents the travel time between population site *i* and provider location *j*.[16]

$$W_{ij}=\{\begin{array}{c} 1,\\ 0.42,\\ 0.03, \end{array}\;\begin{array}{c} if\;t_{ij}\leq 15\;mins\\ if\;15<\;t_{ij}\leq 30\;mins\\ if\;30<\;t_{ij}\leq 60\;mins \end{array}$$

In the first step, for each provider location, the provider to population ratio, *Availability*_*j*_, was calculated by summing the distance decay-weighted population for each ZCTA centroid, *Pop*_*i*_, which falls within the threshold travel time based catchment area.

$$Availability_{j}=\;\frac{N_{Provider}}{\sum_{i\in \left\{t_{ij}\leq t_{thres}\right\}}W_{ij}\left(t_{ij}\right)*\;Pop_{i}}$$

Then, for each ZCTA population weighted centroid, we considered all geocoded provider locations within an initial travel time t_0_ (15 minutes) and summed the provider-to-population ratio (PPR) values for those provider locations within this initial travel time threshold. As was performed in step one, if the summed PPR value was less than 1:3,500, the initial travel time (t_0_) was increased (t_0+15 minutes_) in 15 minute increments until the summed PPR exceeded the 1:3,500 threshold. The travel time at which the PPR threshold was satisfied was used to delineate the healthcare activity catchment area for the ZCTA population weighted centroid. Similar to step one, for 15, 30, and 60 minute drive time catchment areas we applied distance decay weights of 1, 0.42, and 0.03, respectively.

To generate a final measure of provider accessibly for each ZCTA population weighted centroid, all PPRs within the health care activity space were summed applying the distance decay weights.

$$Provider\;Accessibility{\;}_{i}=\;\sum_{j\in \{t_{ij}\leq t_{thres}}W_{ij}\left(t_{ij}\right)*Availability_{j}$$

### Additional data

To explore factors that may be associated with higher spatial accessibility of the various provider groups we collected population data, professional school locations, and U.S. Census designated urbanized area for our analyses.

### U.S. Census and rurality data

Population data were collected at the ZCTA-level from the 2010 U.S. Census. Specifically, we gathered population estimates for percent aged 65 years and older, percent female, median household income, percent under the federal poverty limit, and racial/ethnic composition. U.S. ZCTAs were assigned to states and states were assigned to U.S. Census regions (i.e., Northwest, Midwest, South, West regions) based on if the ZCTA centroids fell within the corresponding geographic boundaries. Provider specific maps for each U.S. Census region are available in the accompanying supporting information (S1–S5 Figs). We also collected rural-urban community area code (RUCA) data that classify each ZCTA as being either “urban”, “large rural”, “small rural”, or “isolated”.[26]

### Professional school data

The location of professional schools is a known factor affecting choice of provider location.[27–29] To develop a roster of accredited professional schools for each provider type, we reviewed accreditation listings from the American Association of Medical Colleges, Association of Colleges of Osteopathic Medicine, the American Association of Colleges of Nursing, and the Association of Chiropractic Colleges. Addresses of accredited professional schools for each healthcare profession were geocoded as point data. The professional schools were aggregated to the ZCTA level. In all cases the professional school location intersected a ZCTA. ZCTAs were then assigned to counties based on if their population weighted centroid intersected a county.

### Statistical analyses

In order to identify spatial clustering of high (and low) spatial accessibility, we calculated the Getis-Ord Gi* statistic for each ZCTA. An optimal fixed distance band analysis was used for conceptualization of spatial relationships. We found the average distance to the 30 nearest neighbors; this distance was approximately 25 miles. The Gi_Bin field was corrected using the FDR correction method for multiple testing and spatial dependence. ZCTAs with a Gi_Bin value of +3 or -3 were statistically significant at the 99% confidence level. We used simple descriptive statistics to examine select population characteristics for clustering of high versus low accessibility areas for each of the provider types.

We calculated Spearman’s rank correlation coefficients (r_s_) to compare spatial accessibility values between provider types. We used generalized linear models to examine associations between population characteristics and spatial accessibility for each provider type. For these analyses the unit of observation was ZCTAs and we assumed a Poisson distribution for the response variable. In our models we examined regional factors and population characteristics. Regional factors included the rurality of the ZCTA (urban, rural, versus isolated) and the presence of a professional school in the county in which the ZCTA was assigned. Population characteristics included sex (percent female), age (percent age 65 and older), minority race/ethnicity (percent all race and ethnicities versus Non-Hispanic White), poverty status (percent under the Federal Poverty Line), and education (percent with less than high school population among those age 25 and older). To improve interpretation of coefficients, we collapsed population age, minority race/ethnicity, poverty status, and education into terciles (representing low, medium, and high levels respectively). Coefficients were expressed as rate ratios and robust estimation methods were used to correct standard errors for deviation in response distributional assumption. P-values were adjusted for false discovery rate (FDR) to correct for multiple hypothesis tests.[30]

**Fig 1:**
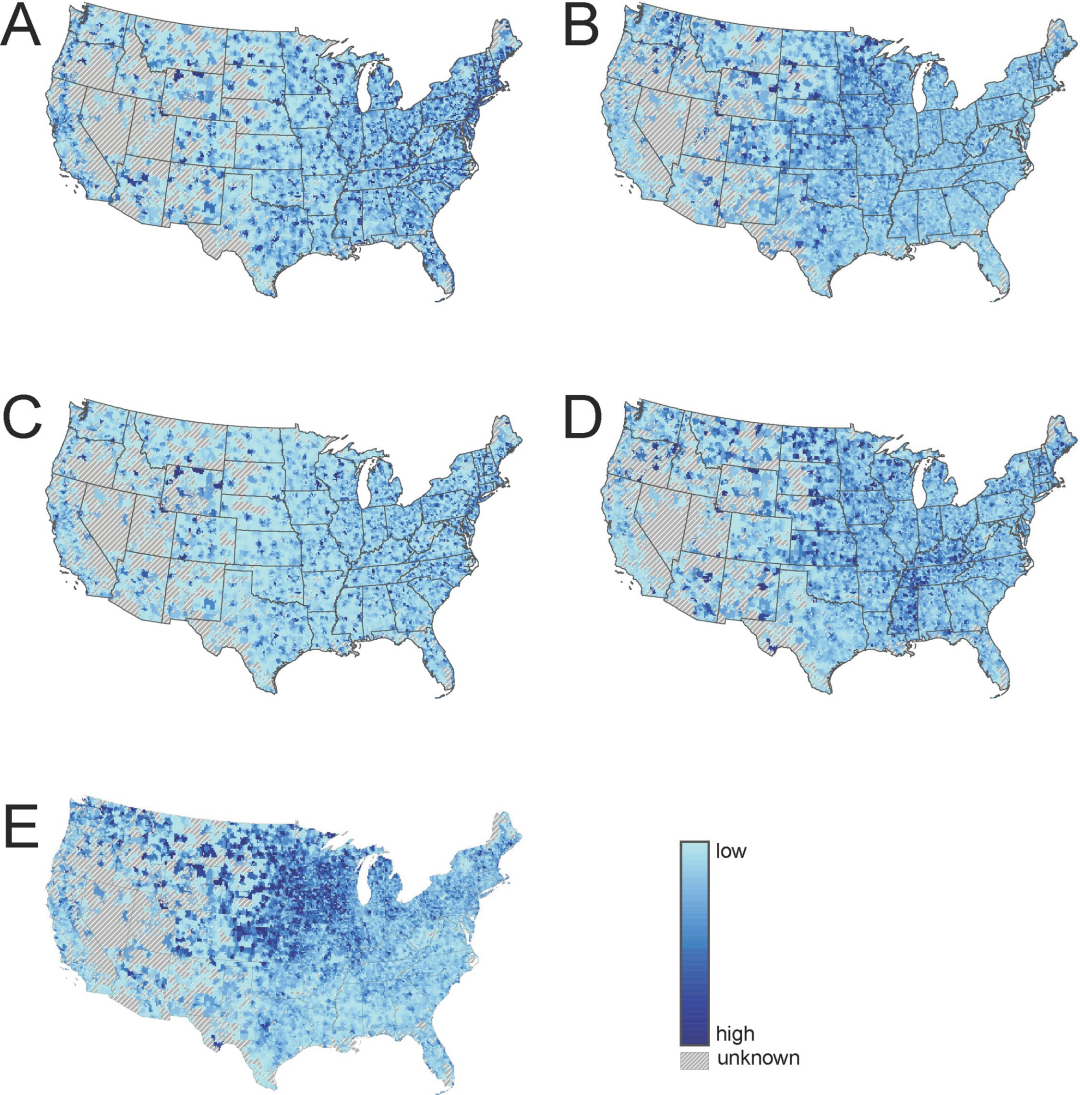
Provider spatial accessibility. Accessibility for internal medicine physicians (A), family medicine physicians (B), specialists (C), nurse practitioners (D), and chiropractors (E) across the contiguous United States.

**Fig 2:**
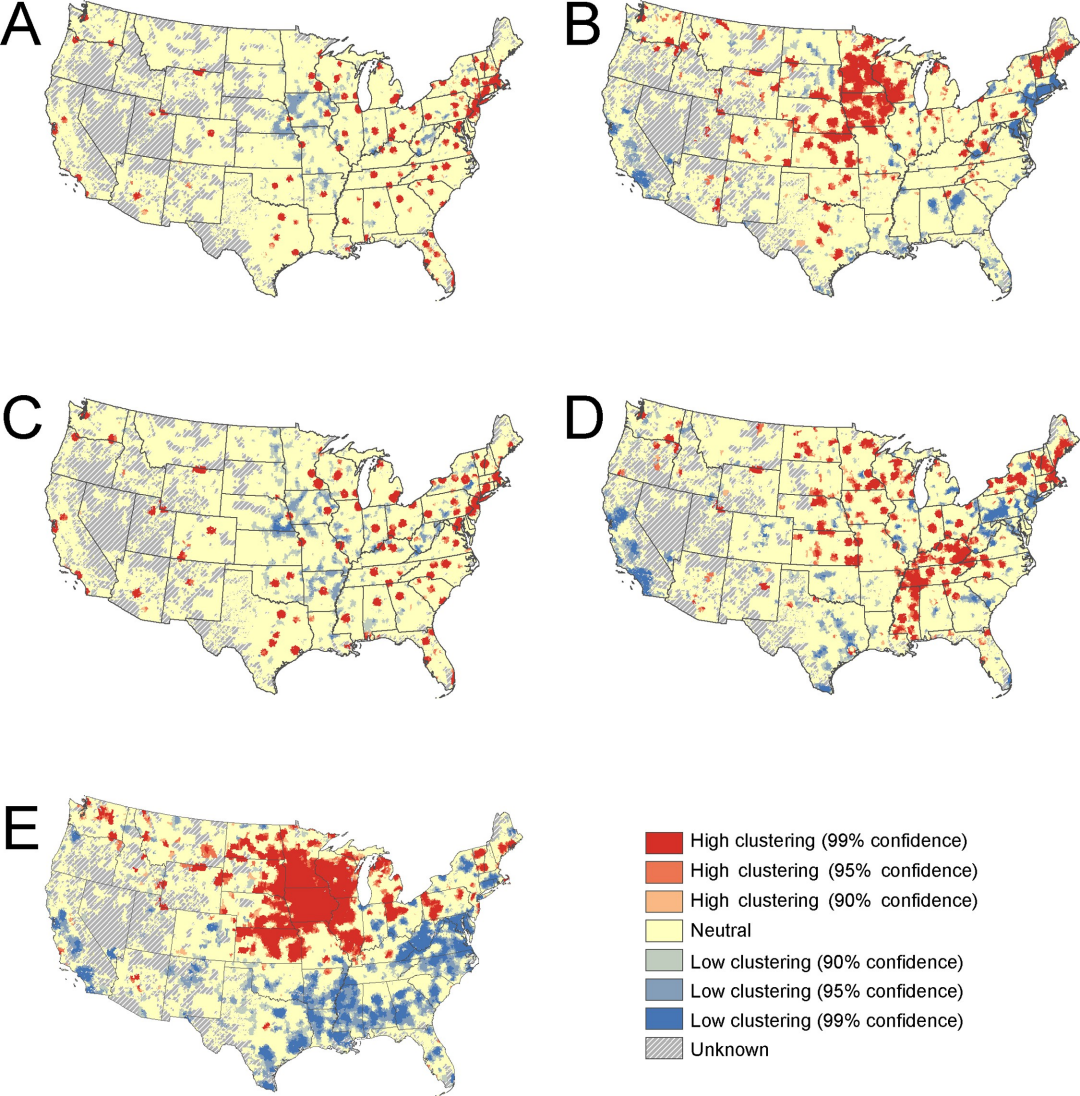
Provider Getis-Ord Gi* statistic. Getis-Ord Gi* statistic for internal medicine physicians (A), family medicine physicians (B), specialists (C), nurse practitioners (D), and chiropractors (E) across the contiguous United States.

Geospatial analyses were conducted using ESRI ArcGIS, version 10.5 and StreetMap Premium, 2014 version 1 (Redlands, CA). U.S. Census Tiger Cartographic Boundary datasets were used in Figs 1 and 2 and S1–S5 [31, 32] Analysis of NPPES and claims data were conducted using SAS, version 9.4 (Cary, NC). All analyses were based on complete case analysis and we assumed any missing values to be missing completely at random.

## Results

### Provider spatial accessibility

Using the combination of data from the NPPES and Medicare claims we identified 178,660 internal medicine physicians, 90,870 family medicine physicians, 391,621 specialists, 79,790 nurse practitioners, and 44,040 chiropractors who were actively treating patients in 2014. The distribution of spatial accessibility nationally by provider type is demonstrated in Fig 1. Overall, for each provider type, spatial accessibility was not evenly distributed across ZCTAs. For example, in the case of family medicine physicians, (Fig 1B) an area of high spatial accessibility was concentrated in the Midwestern U.S., which is visually represented by the darker shading of ZCTAs, while the lighter shading observed across the western and southern U.S. represents ZCTAs with comparatively lower spatial accessibility values for family medicine physicians relative to other areas. While, in the case of internal medicine physicians and specialists, a higher spatial accessibility was observed in the Northeast Region. For nurse practitioners, spatial accessibility was lowest in the West and uniform across the Midwest, South, and Northeast regions. Lastly, among chiropractors, high spatial accessibility was observed in the Midwest Region and relatively lower spatial accessibility in the South and West regions.

### Getis-Ord Gi* analyses

Getis-Ord Gi* analysis revealed unique patterns of higher and lower spatial accessibility among the different provider types. More specifically, family medicine physicians were observed to have clusters of high spatial accessibility that were spatially distinct in comparison to those seen for internal medicine physicians (r_s_ = 0.2693, p < 0.001). Although there were clusters of high spatial accessibility distributed across the U.S., areas of family medicine physician high spatial accessibility were concentrated in the Midwestern U.S., with low spatial accessibility clusters located in the Northwestern U.S. and along the west coast (Fig 2B). In contrast, internal medicine physicians had high spatial accessibility clusters with locations that seemed to correspond with U.S. Census Bureau-designated urbanized areas (Fig 2A). This can be more easily visualized on the provider specific regional maps (see S1–S5 Figs). This pattern of high spatial accessibility for internal medicine physicians near urbanized areas was similar to that of specialists (r_s_ = 0.8082, p < 0.001).

The spatial accessibility of nurse practitioners displayed a somewhat unique pattern that was most similar to that of family medicine physicians (r_s_ = 0.4444, p < 0.001). Clusters of high spatial accessibility for nurse practitioner were observed along the Mississippi River Valley, Northeastern U.S., and the Midwest. However, there was a pattern of low spatial accessibility for nurse practitioners in the West. While chiropractors were noted to have a large cluster of high spatial accessibility in the Midwest, low spatial accessibility clusters were observed in the South and West.

### Provider spatial accessibility and population characteristics

The characteristics of populations that live within the areas of high and low spatial accessibility also differed by provider group, Table 2. In our adjusted analyses independent factors associated with higher spatial accessibility were very similar between internal medicine physicians and specialists–presence of a medical school in the county was associated with approximately 70% higher spatial accessibility and higher spatial accessibility was associated with urban locales, Table 3. Higher spatial accessibility of these two provider groups was also associated with higher racial/ethnicity diversity and poverty. For instance among specialists, spatial accessibility was approximately 40% higher in high poverty areas compared to low (RR 1.43, 95% CI: 1.37, 1.49). Family medicine physicians and nurse practitioners were similar to each other in regards to predictors of higher spatial accessibility–higher spatial accessibility was associated with rural locales and less racial/ethnic diversity. Among all provider types, family medicine was the only provider type with higher spatial accessibility in isolated areas (as compared to urban). Chiropractors were somewhat unique, with higher spatial accessibility being associated with less racial/ethnic diversity, lower poverty, and a higher percentage of older adults.

**Table 2:** Population characteristics within areas of high, neutral, and low spatial accessibility, by provider type.

	Total Population	% Female, mean	% Population age 65+, mean	Median household income, mean	% Poverty, mean	% Non-Hispanic White, mean	% Non-Hispanic Black, mean	% Hispanic, mean	% Other race/ethnicity, mean
Family medicine physicians									
Low	102,165,392	51.1	13.0	$63,265	14.3	47.4	15.3	25.8	11.5
Neutral	179,196,982	50.7	14.6	$47,182	15.8	68.6	11.4	14.0	6.0
High	31,599,871	50.8	14.7	$51,248	13.0	77.3	8.1	7.0	7.6
Internal medicine physicians									
Low	3,711,846	50.1	17.5	$43,517	15.2	87.9	3.4	5.5	3.2
Neutral	153,329,131	50.4	14.8	$46,579	15.7	68.9	9.4	15.9	5.8
High	155,427,165	51.3	13.3	$65,247	13.3	55.6	15.6	18.7	10.1
Specialists									
Low	6,173,070	49.9	17.3	$41,977	16.2	82.5	7.5	6.1	3.9
Neutral	143,786,129	50.4	15.2	$46,546	15.6	69.4	9.3	15.6	5.7
High	162,827,206	51.2	13.0	$65,154	13.2	55.7	15.3	18.9	10.1
Nurse practitioners									
Low	88,398,667	50.8	13.2	$59,547	14.3	47.1	11.1	29.7	12.1
Neutral	154,433,484	50.7	14.8	$47,621	15.4	67.8	12.5	13.7	6.0
High	70,055,356	51.1	13.8	$52,584	14.9	70.6	13.6	8.9	6.9
Chiropractors									
Low	157,728,674	51.0	12.7	$51,632	17.1	50.6	16.5	23.3	9.7
Neutral	115,868,215	50.7	15.5	$49,215	15.0	72.2	9.0	12.4	6.4
High	39,443,803	50.5	15.6	$52,295	12.1	82.0	5.7	6.8	5.6

Low represent areas of low clustering at 99% confidence level, Neutral represents areas with no significant clustering, and High represents areas of high clustering at 99% confidence level. All population characteristics were taken from the 2010 US Census.

**Table 3:** Adjusted rate ratios for the association between population characteristics and provider spatial accessibility.

	Primary Care Physicians				
Independent Variable	Internal Medicine	Family Medicine	Specialist	Nurse Practitioner	Chiropractor
Presence of professional school^a^	1.71 (1.65, 1.77)*	1.04 (1.02, 1.07)*	1.73 (1.67, 1.79)*	1.29 (1.25, 1.33)*	1.00 (0.95, 1.07)
Urban or rural area					
Urban	1.00 (reference)	1.00 (reference)	1.00 (reference)	1.00 (reference)	1.00 (reference)
Large rural	0.83 (0.80, 0.88)*	1.18 (1.13, 1.23)*	0.93 (0.88, 0.98)*	1.18 (1.13, 1.23)*	1.25 (1.20, 1.29)*
Small rural	0.52 (0.50, 0.55)*	1.39 (1.34, 1.44)*	0.56 (0.54, 0.59)*	1.04 (1.00, 1.07)	1.37 (1.32, 1.41)*
Isolated	0.44 (0.41, 0.47)*	1.18 (1.15, 1.21)*	0.42 (0.40, 0.45)*	0.98 (0.95, 1.01)	1.19 (1.15, 1.22)*
Population characteristics					
Percent female	1.00 (1.00, 1.01)*	1.00 (1.00, 1.00)	1.00 (1.00, 1.01)*	1.00 (1.00, 1.01)*	1.00 (1.00, 1.00)
Population age 65+^b^					
Low	1.00 (reference)	1.00 (reference)	1.00 (reference)	1.00 (reference)	1.00 (reference)
Medium	1.06 (1.03, 1.10)*	1.02 (0.99, 1.05)	1.04 (1.00, 1.08)*	1.02 (0.99, 1.05)	1.07 (1.04, 1.10)*
High	0.98 (0.95, 1.02)	0.98 (0.95, 1.01)	0.94 (0.90, 0.98)*	0.94 (0.91, 0.97)*	1.05 (1.02, 1.07)*
Minority race/ethnic population^b^^,^^c^					
Low	1.00 (reference)	1.00 (reference)	1.00 (reference)	1.00 (reference)	1.00 (reference)
Medium	1.13 (1.08, 1.17)*	0.97 (0.95, 1.00)	1.16 (1.11, 1.21)*	0.99 (0.96, 1.02)	0.89 (0.86, 0.91)*
High	1.28 (1.23, 1.33)*	0.87 (0.85, 0.89)*	1.27 (1.22, 1.33)*	0.86 (0.83, 0.89)*	0.62 (0.60, 0.64)*
Poverty population^b^^,^^d^					
Low	1.00 (reference)	1.00 (reference)	1.00 (reference)	1.00 (reference)	1.00 (reference)
Medium	1.10 (1.06, 1.14)*	1.10 (1.07, 1.12)*	1.13 (1.09, 1.18)*	1.13 (1.10, 1.12)*	1.01 (0.98, 1.03)
High	1.37 (1.31, 1.43)*	1.10 (1.08, 1.13)*	1.43 (1.37, 1.49)*	1.39 (1.34, 1.44)*	0.86 (0.84, 0.89)*
Population < high school education^b^^,^^e^					
Low	1.00 (reference)	1.00 (reference)	1.00 (reference)	1.00 (reference)	1.00 (reference)
Medium	0.71 (0.68, 0.74)*	0.99 (0.96, 1.01)	0.70 (0.67, 0.72)*	0.88 (0.86, 0.91)*	1.00 (0.97, 1.02)
High	0.60 (0.58, 0.63)*	0.82 (0.80, 0.84)*	0.56 (0.54, 0.59)*	0.76 (0.73, 0.78)*	0.75 (0.72, 0.77)*

Abbreviations: CI, confidence interval

a: Presence of corresponding professional school (e.g., medical school) in county

b: Population characteristics defined as "low", "medium", versus "high" based terciles (e.g., percent of the ZCTA population age 65 and older collapsed into terciles)

c: Minority race/ethnic population based on percent minority group (i.e., Non-Hispanic Black, Hispanic, and other) versus Non-Hispanic White

d: ZCTA poverty status based on percent of the population living below the Federal Poverty Level

e: Population with less than a high school based on the ZCTA population age 25 and older

* Indicates statistically significant based on correction for False Discovery Rate (FDR).

## Discussion

The objective of our study was to compare spatial accessibility of different healthcare provider types using current state of the art geospatial methodology and to examine factors associated with higher spatial accessibility. To our knowledge this is the first study to examine spatial accessibility at the ZCTA level using the VE2SFCA method across the contiguous U.S. Overall, we found spatial accessibility was not equally distributed across geographic areas among all of the five provider types examined–each were found to have distinct areas of concentrated high (and low) spatial accessibility. Most notably, we found that despite both being considered a “primary care physician”, spatial accessibility differed considerably between internal medicine and family medicine physicians (r_s_ = 0.2693, p < 0.001). Internal medicine physicians more resembled specialists, being more likely to be in condensed urban locales and strongly associated with the presence of a medical school (r_s_ = 0.8082, p < 0.001).

Maldistribution of the healthcare workforce has been a widely recognized problem since the publication of the Graduate Medical Education National Advisory Committee report in 1980.[33] Since that time, multiple studies have demonstrated the substantial variation in geographic accessibility to physicians. However, previous studies may have masked small-area variation in accessibility, due to their reliance on measuring accessibility using county, state, or even regional area units.[11, 34–37] While studies that measured small-areas were limited in their scale to examining specific cities, states, regions, or populations.[12, 14, 17, 20, 21] The VE2SFCA method we used has several advantages over traditional provider-to-population ratios (i.e., “per capita”), which rely on administrative borders as the unit of analysis. Due to use of drive-time as a distance related impedance measure, the VE2SFCA method is less dependent on the aggregation of data into polygon-based administrative borders such as counties, cities, or ZIP codes. The VE2SFCA method also allowed us to examine small-area variation at the ZCTA level on a national scale.

Application of the VE2SFCA method to national data on practice location revealed considerable differences. Internal medicine physicians had the highest spatial accessibility in population dense areas and spatial accessibility was associated with higher poverty and greater proportions of non-Hispanic black and Hispanic individuals. Associations between nurse practitioner spatial accessibility was highest in areas with an intermediate population density and racial/ethnic diversity, while family medicine physicians were most accessible in areas with the comparatively lowest population density and racial diversity. As a provider group outside of traditional medicine, chiropractors were the most unlike other provider types in regards to both their spatial accessibility pattern and their predictors of higher spatial accessibility.

Examining spatial accessibility of non-physicians (i.e., nurse practitioners and chiropractors) is a particular strength of our study. Advanced practice providers, including nurse practitioners, are playing an increasingly important role in healthcare delivery. Our analyses demonstrate that nurse practitioners share some similarities to other groups yet have some distinct differences which may hint at providing care in underserved areas. While family medicine physicians and nurse practitioners shared some common predictors such as rural locales, their patterns were spatially distinct at the national scale–family medicine physicians had higher spatial accessibility in the upper Midwest whereas nurse practitioners had higher spatial accessibility in the South. Furthermore, unlike the predictors of spatial accessibility for family medicine physicians, nurse practitioner spatial accessibility was not higher in small or isolated rural areas compared to urban areas. This suggests that currently there may be workforce supply limits to the use of nurse practitioners to supplement physicians in these community areas.

## Limitations

There are several limitations that should be considered when interpreting the study findings. First, healthcare access represents more than spatial accessibility alone.[19] The concept of access also includes acceptability (patient attitudes and beliefs), accommodation (wait times, provider workload), affordability (cost, insurance coverage), and availability (treatments and services offered).[38] We chose to examine spatial accessibility because it is the fundamental requirement for the other components of access. Second, the VE2SFCA method assumes that all providers and populations that are located within a drive-time based catchment area have equal accessibility. We cede that even within small areas, healthcare accessibility is inequitable. For this reason, we have included sociodemographic population characteristics in the analysis, such as: age, sex, median household income, poverty level, and race/ethnicity. Third, a single PPR of 1:3,500 was used as the threshold value. We selected this value due to its real world use in defining primary care related health professional shortage areas by the U.S. Department of Health and Human Services. Ratios below this value are not felt to be adequate for providing primary care medical services. Forth, discrete distance decay weights were applied to differentiate travel time zones across provider types instead of a continuous function. To properly apply differing distance decay functions to each provider type, patient specific data of actual utilization of health services for each provider type would be necessary.[18] Fifth, linear models examining associations between population characteristics and PRP utilized cross-sectional data and therefore their findings represent associations and we cannot rule out reverse causality. Sixth, provider practice locations were determined based on data from the NPPES and practice addresses were not confirmed for their accuracy. However, in a recent comparison study, the NPPES had the highest accuracy for provider contact information in comparison to other commonly used national sources such as the American Medical Association Physician Masterfile and the SK&A file.[39] Lastly, some sparsely populated areas did not contain healthcare providers or sufficient numbers of residents to be included in the study analysis. These areas are typically located in small rural or remote frontier communities and their representation within the study may be underreported.

## Conclusions

Disparities in access to primary care services greatly impacts population health.[34, 40] Through use of the VE2SFA method, we have estimated spatial accessibility to primary care providers on a national scale, at ZCTA-level resolution. Unlike, per-capita based provider-to-population rations, VE2SFA spatial accessibility measurements employ dynamic, drive-time based, catchment areas that incorporate population thresholds and an estimation of distance decay in utilization. Our findings indicate that the primary care workforce is unequally distributed across the nation, with internal medicine physicians, family medicine physicians, and nurse practitioners each displaying a unique pattern for their spatial accessibility. The characteristics of populations that live within the areas of high and low spatial accessibility also differed by provider type. In light of these findings, future programs and policies intended to address maldistribution of the primary care workforce may need to be individualized according to provider type, target population, and geographic location. Additional research is needed to explore the factors that influence geographic patterns of spatial accessibility and the interaction between primary care provider groups.

## Supporting information

S1 Fig Internal medicine physician accessibility and Getis-Ord Gi* statistic by U.S. census region. (PDF)

S2 Fig Family medicine physician accessibility and Getis-Ord Gi* statistic by U.S. census region. (PDF)

S3 Fig Specialist physician accessibility and Getis-Ord Gi* statistic by U.S. census region. (PDF)

S4 Fig Nurse practitioner accessibility and Getis-Ord Gi* statistic by U.S. census region. (PDF)

S5 Fig Chiropractor accessibility and Getis-Ord Gi* statistic by U.S. census region. (PDF)

S1 File Minimum data set. (CSV)
