# Supplementary material for: Geographic variation in spatial accessibility of U.S. healthcare providers
Source: PLoS One. 2019 Apr 9;14(4):e0215016. doi: 10.1371/journal.pone.0215016 (PMC6456202; doi:10.1371/journal.pone.0215016)
Supplement: S2 Fig — (PDF) [file pone.0215016.s002.pdf]

Family medicine physician accessibility and Getis-Ord Gi* statistic by U.S. census region

Fig 2.1. Spatial accessibility for family medicine physicians (A) and Getis-Ord Gi* statistic (B) in the Northeast.

Fig 2.2. Spatial accessibility for family medicine physicians (A) and Getis-Ord Gi* statistic (B) in the Midwest.

Fig 2.3. Spatial accessibility for family medicine physicians (A) and Getis-Ord Gi* statistic (B) in the South.

Fig 2.4. Spatial accessibility for family medicine physicians (A) and Getis-Ord Gi* statistic (B) in the West.

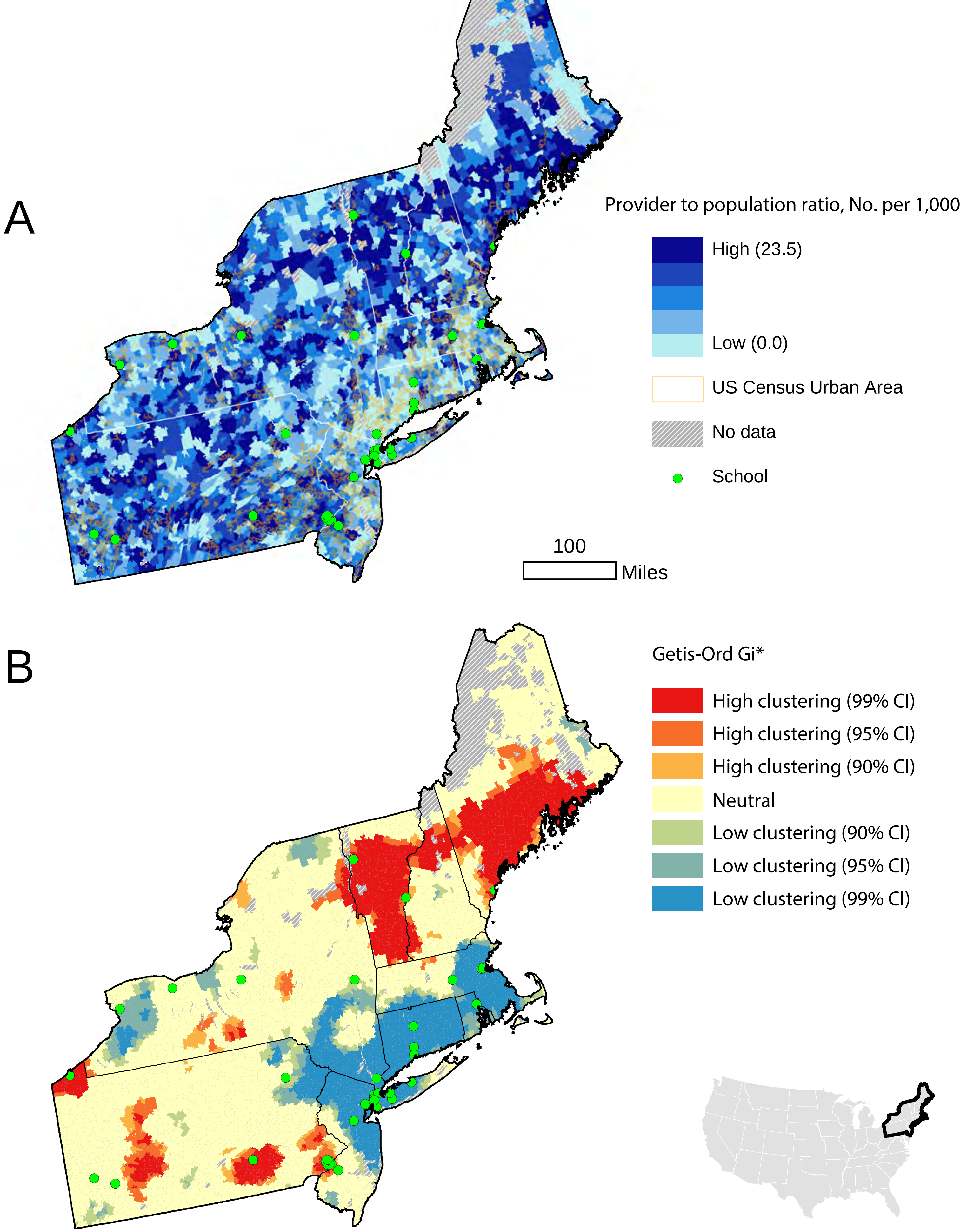

Figure 2.1. Provider to population ratio for family medicine physicians (A) and Getis-Ord Gi* statistic (B) in the

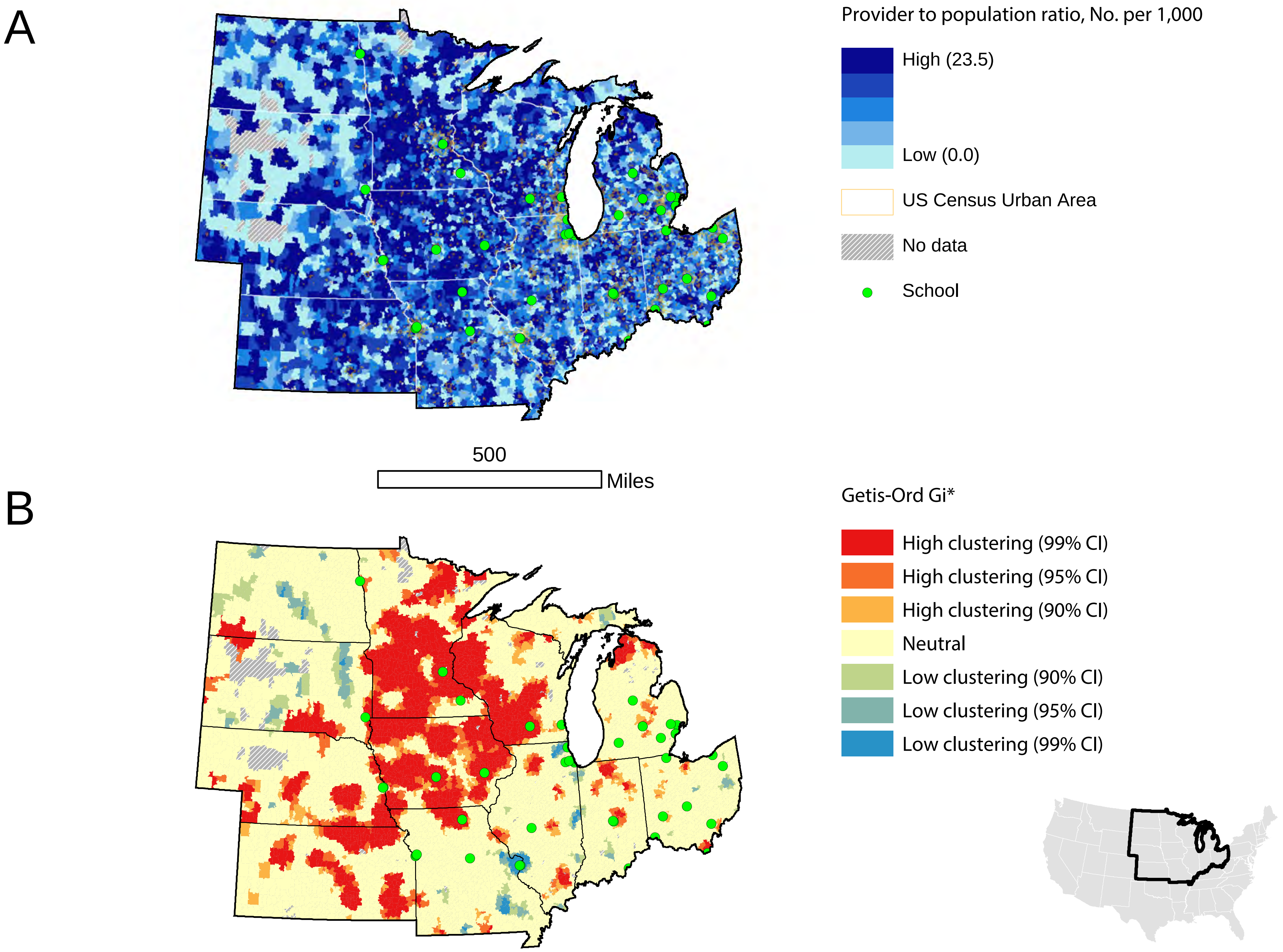

Figure 2.2. Provider to population ratio for family medicine physicians (A) and Getis-Ord Gi* statistic (B) in the Midwest.

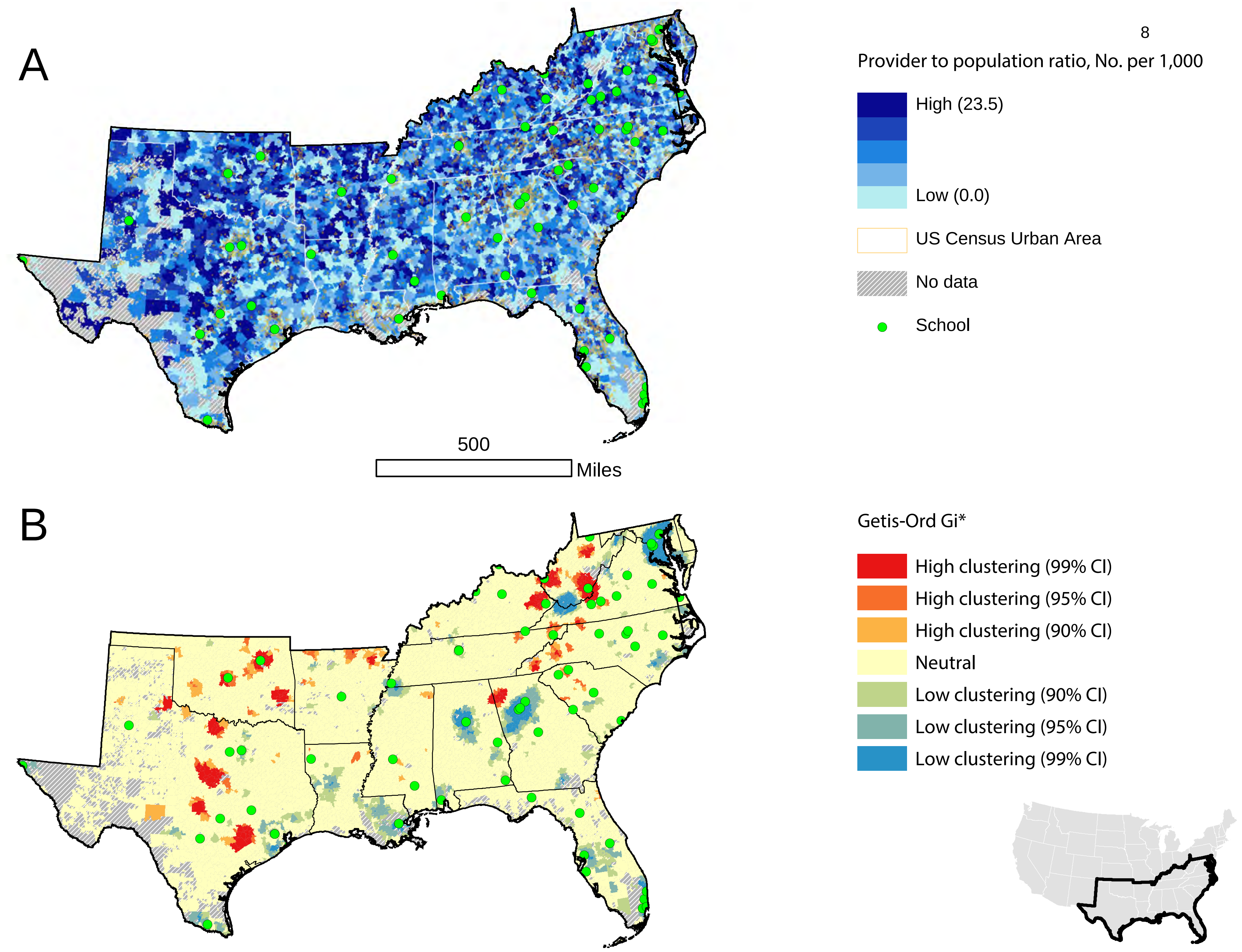

Figure 2.3. Provider to population ratio for family medicine physicians (A) and Getis-Ord Gi* statistic (B) in the South.

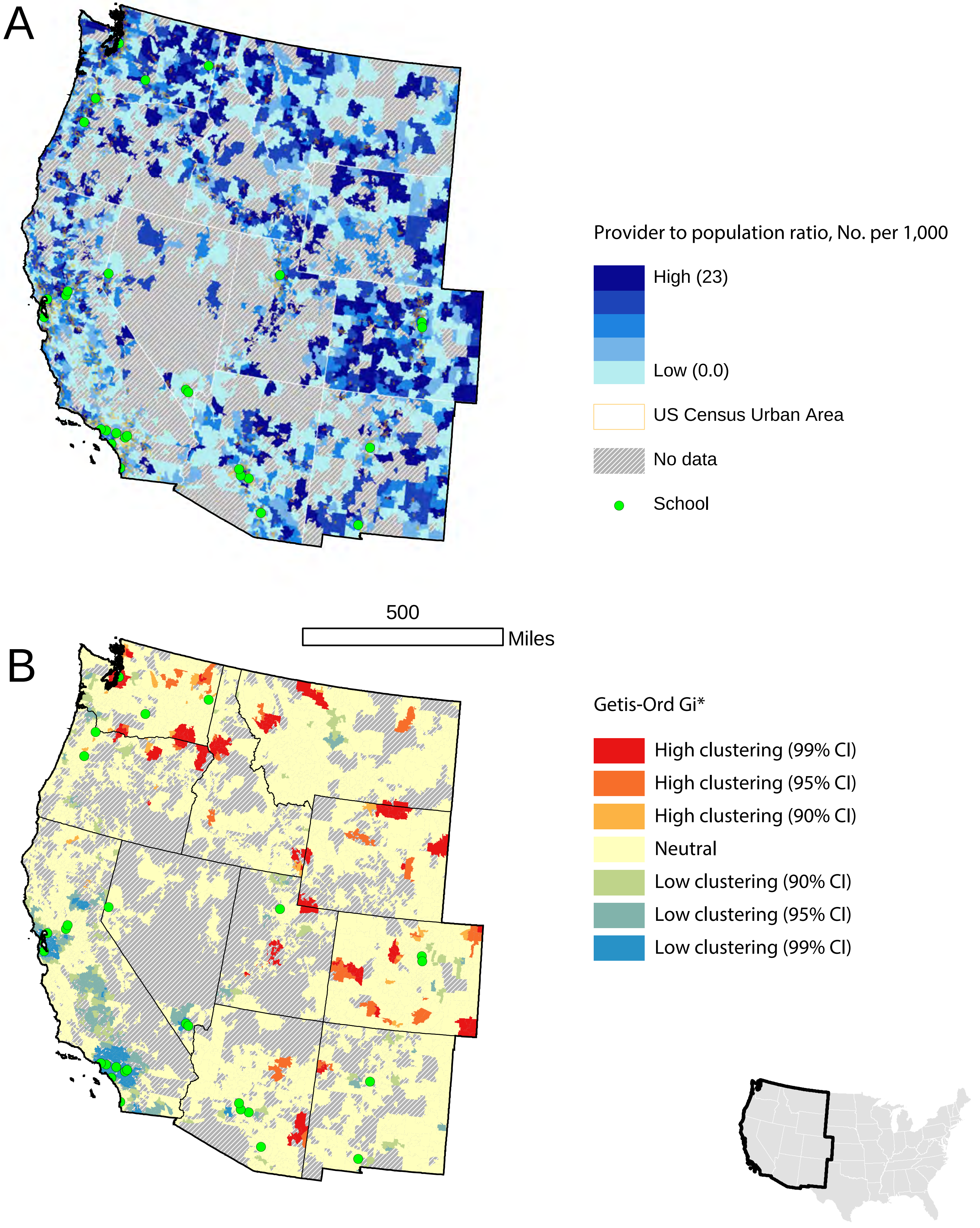

Figure 2.4. Provider to population ratio for family medicine physicians (A) and Getis-Ord Gi* statistic (B) in the West.
