# Supplementary material for: Geographic variation in spatial accessibility of U.S. healthcare providers
Source: PLoS One. 2019 Apr 9;14(4):e0215016. doi: 10.1371/journal.pone.0215016 (PMC6456202; doi:10.1371/journal.pone.0215016)
Supplement: S3 Fig — (PDF) [file pone.0215016.s003.pdf]

Specialist physician accessibility and Getis-Ord Gi* statistic by U.S. census region

Fig 3.1. Spatial accessibility for specialists (A) and Getis-Ord Gi* statistic (B) in the Northeast.

Fig 3.2. Spatial accessibility for specialists (A) and Getis-Ord Gi* statistic (B) in the Midwest.

Fig 3.3. Spatial accessibility for specialists (A) and Getis-Ord Gi* statistic (B) in the South.

Fig 3.4. Spatial accessibility for specialists (A) and Getis-Ord Gi* statistic (B) in the West.

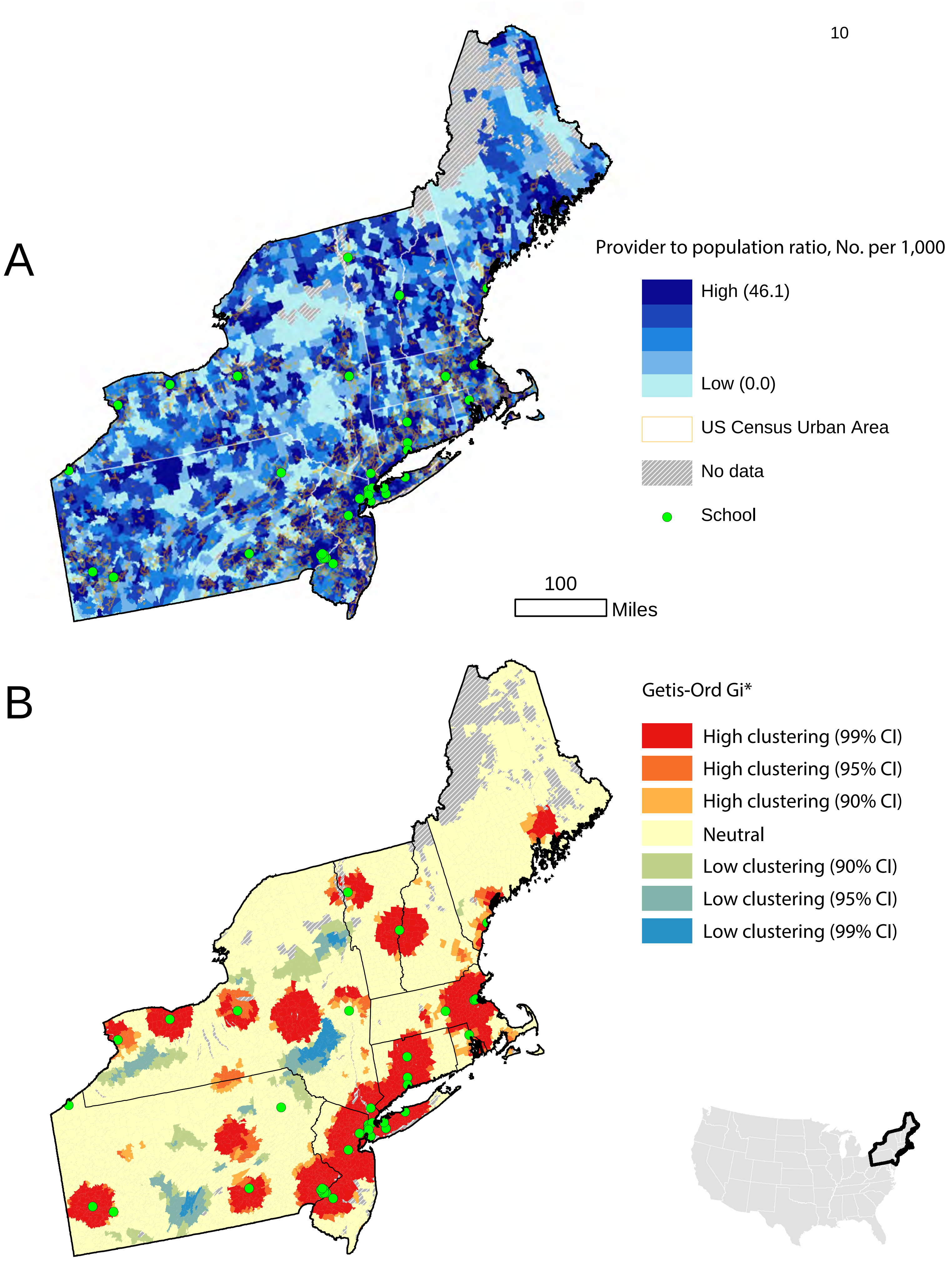

Figure 3.1. Provider to population ratio for specialists (A) and Getis-Ord Gi* statistic (B) in the Northeast.

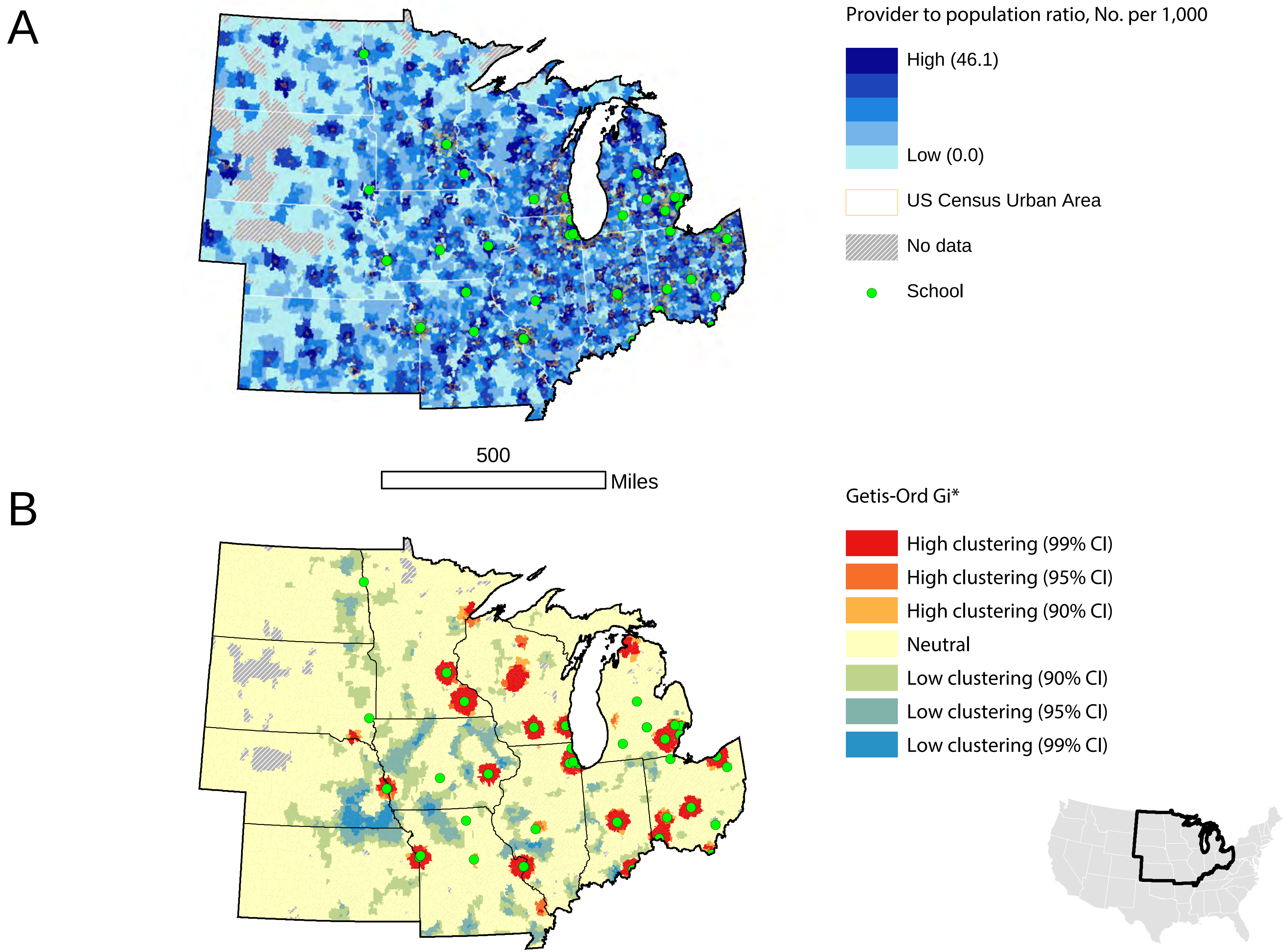

Figure 3.2. Provider to population ratio for specialists (A) and Getis-Ord Gi* statistic (B) in the Midwest.

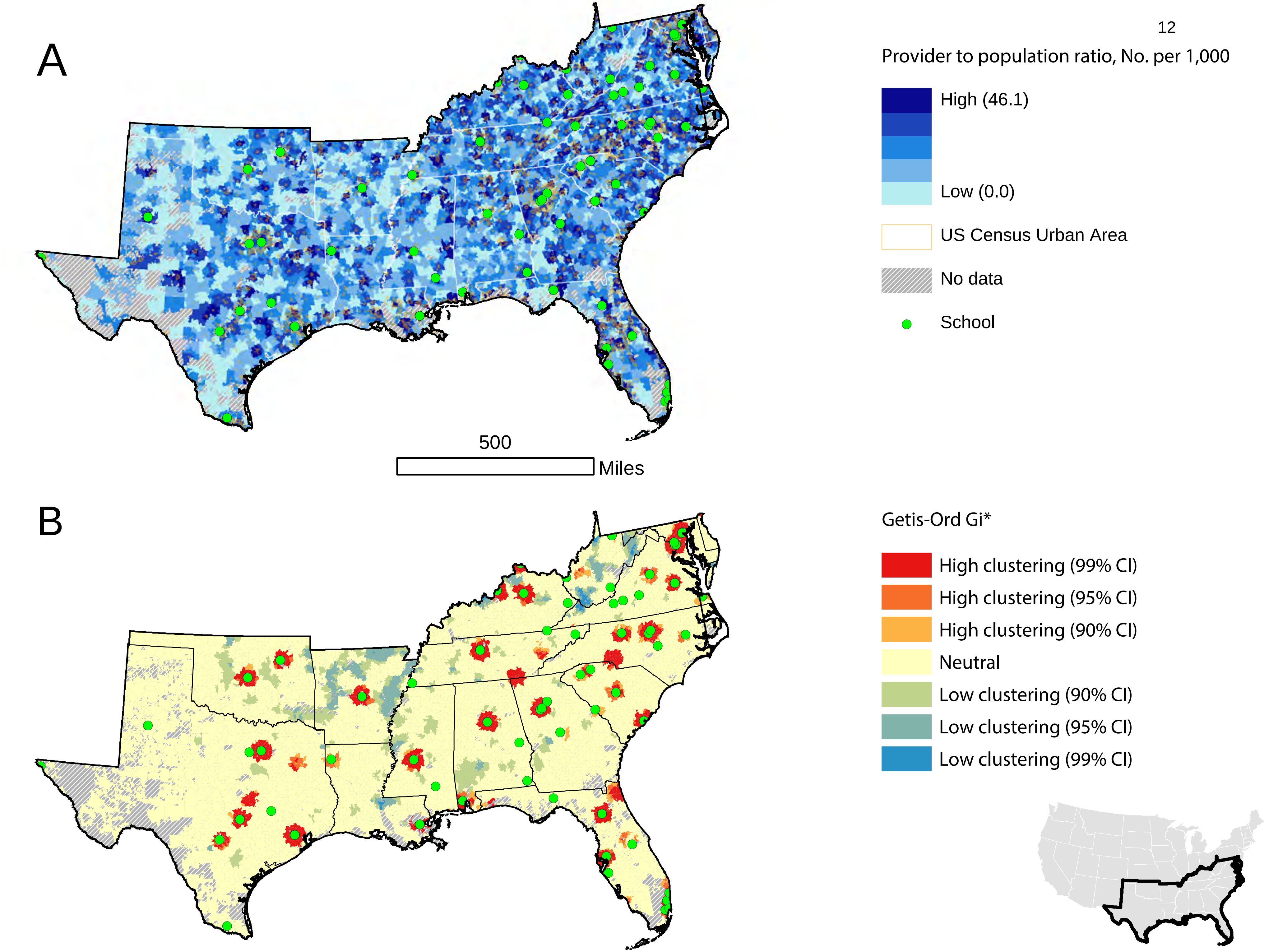

Figure 3.3. Provider to population ratio for specialists (A) and Getis-Ord Gi* statistic (B) in the South

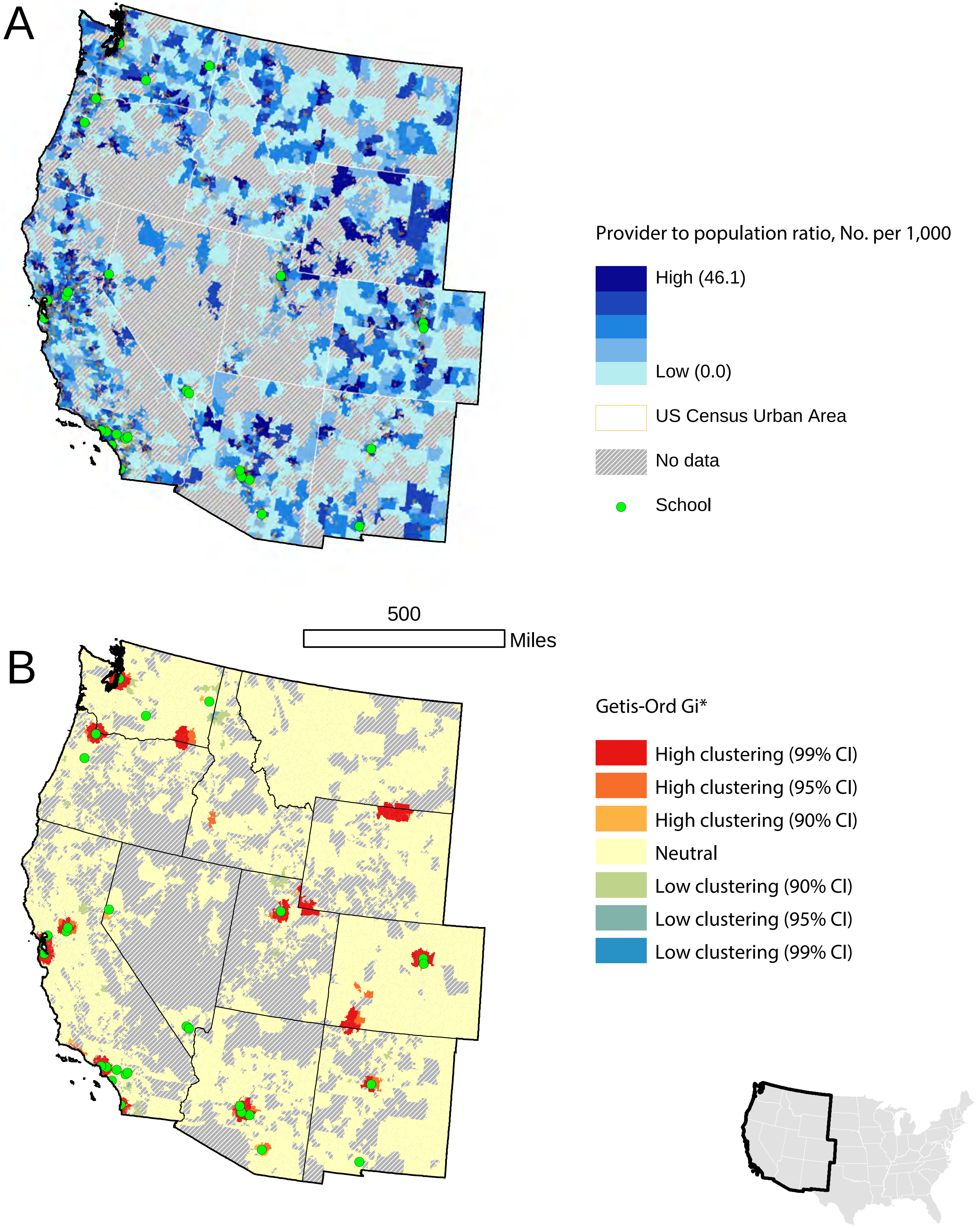

Figure 3.4. Provider to population ratio for specialists (A) and Getis-Ord Gi* statistic (B) in the West.
