# Supplementary material for: Geographic variation in spatial accessibility of U.S. healthcare providers
Source: PLoS One. 2019 Apr 9;14(4):e0215016. doi: 10.1371/journal.pone.0215016 (PMC6456202; doi:10.1371/journal.pone.0215016)
Supplement: S4 Fig — (PDF) [file pone.0215016.s004.pdf]

Nurse Practitioner accessibility and Getis-Ord Gi* statistic by U.S. census region

Fig 4.1. Spatial accessibility for nurse practitioners (A) and Getis-Ord Gi* statistic (B) in the Northeast.

Fig 4.2. Spatial accessibility for nurse practitioners (A) and Getis-Ord Gi* statistic (B) in the Midwest.

Fig 4.3. Spatial accessibility for nurse practitioners (A) and Getis-Ord Gi* statistic (B) in the South.

Fig 4.4. Spatial accessibility for nurse practitioners (A) and Getis-Ord Gi* statistic (B) in the West.

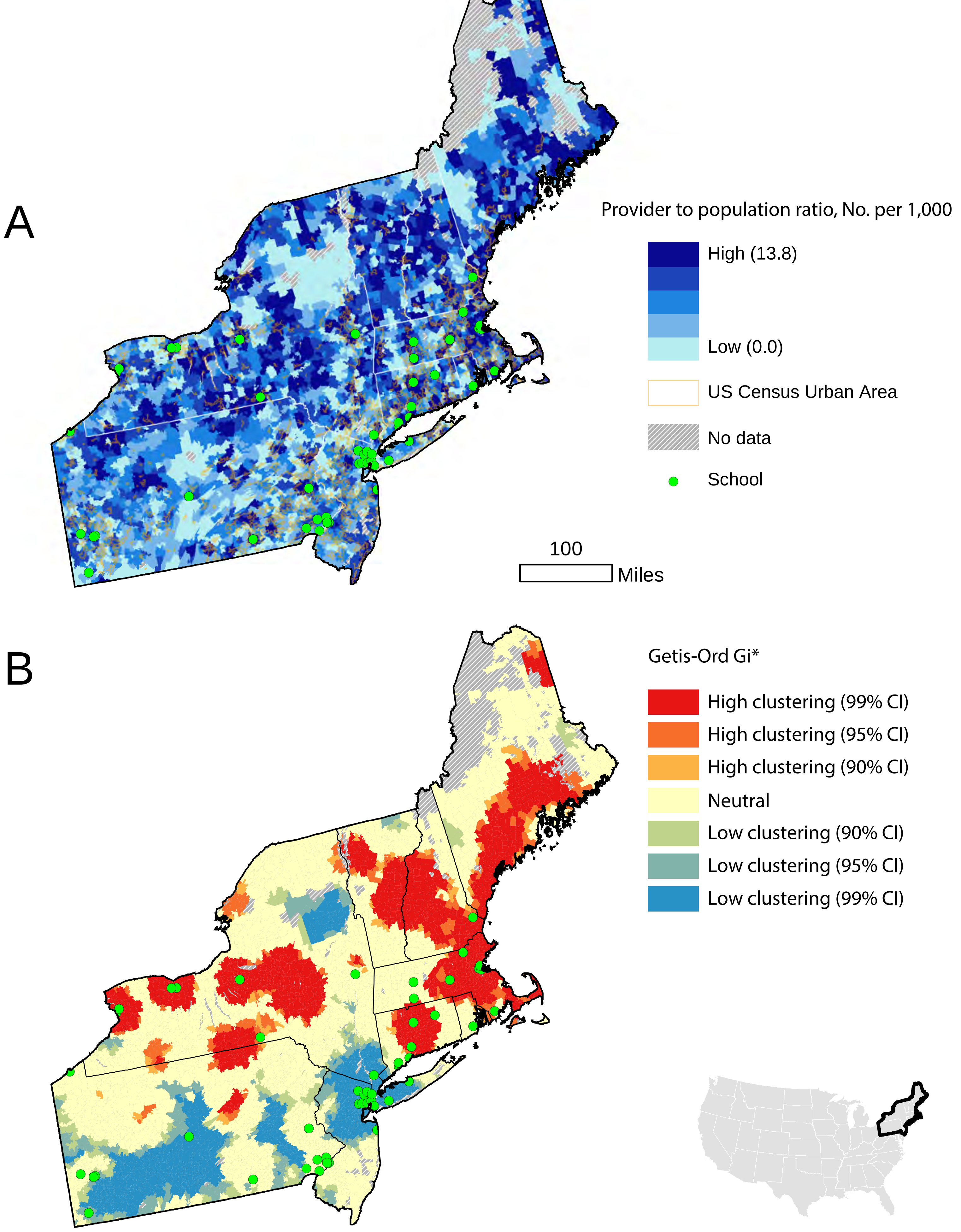

Figure 4.1. Provider to population ratio for nurse practitioners (A) and Getis-Ord Gi* statistic (B) in the Northeast.

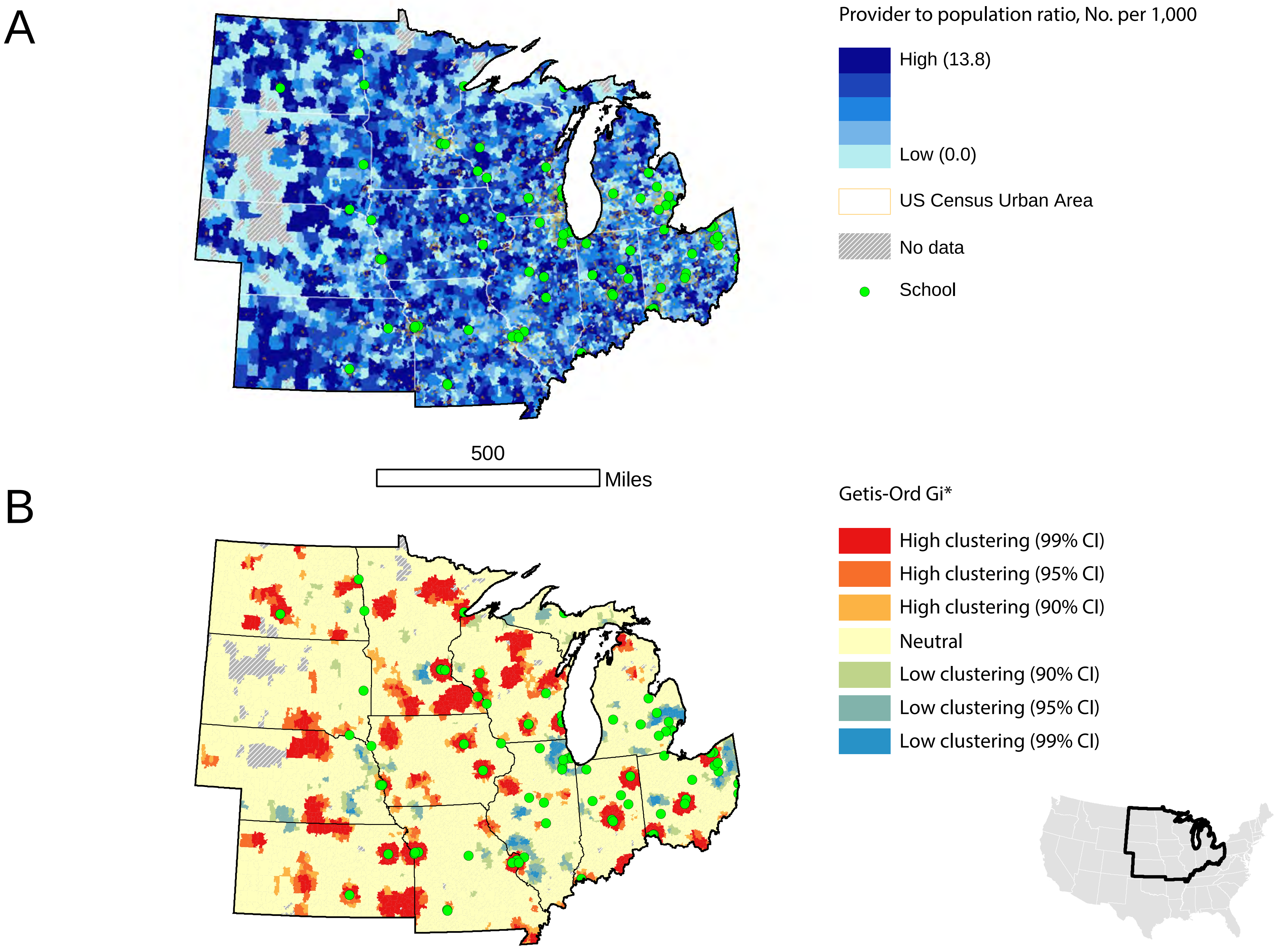

Figure 4.2. Provider to population ratio for nurse practitioners (A) and Getis-Ord Gi* statistic (B) in the Midwest.

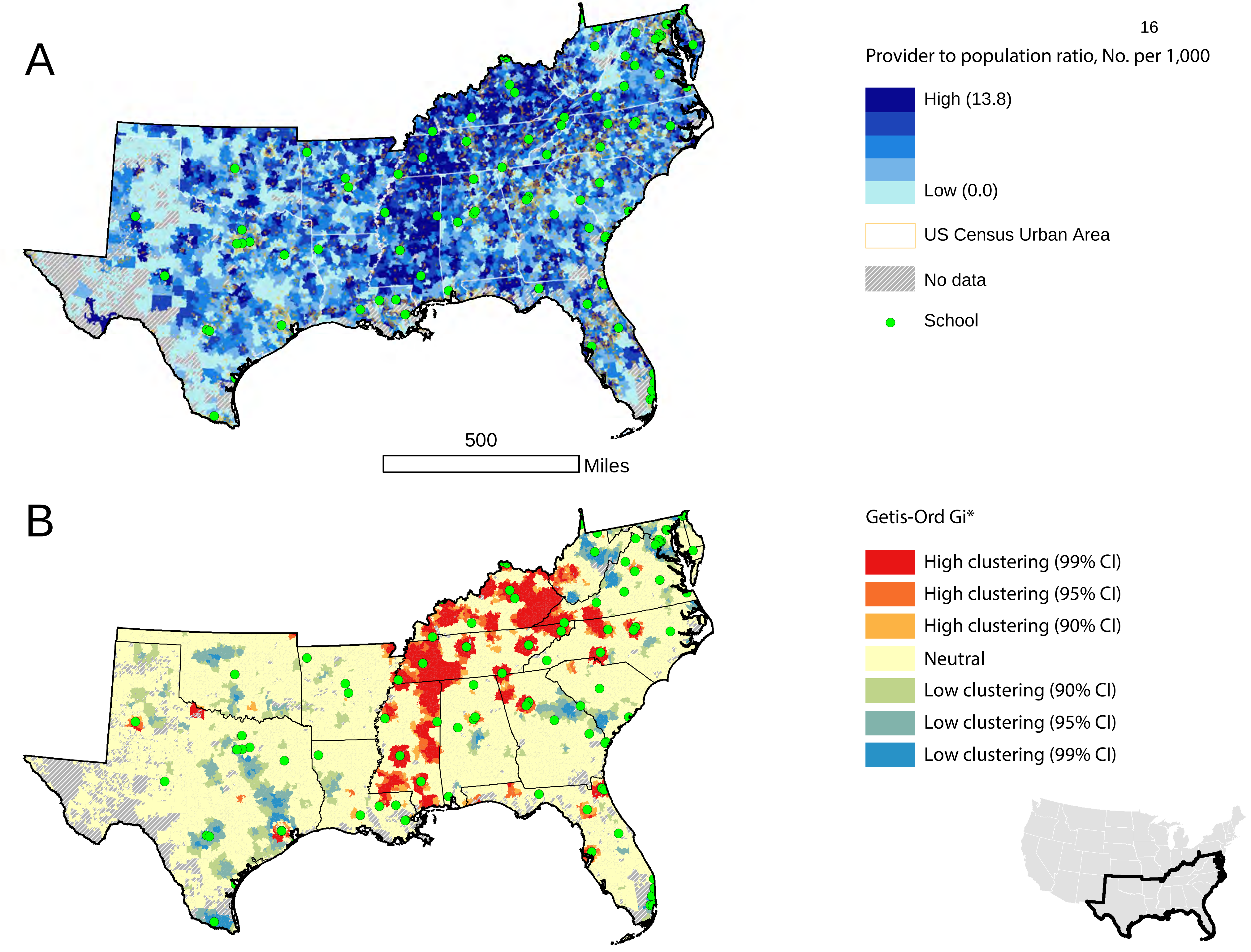

Figure 4.3. Provider to population ratio for nurse practitioners (A) and Getis-Ord Gi* statistic (B) in the South.

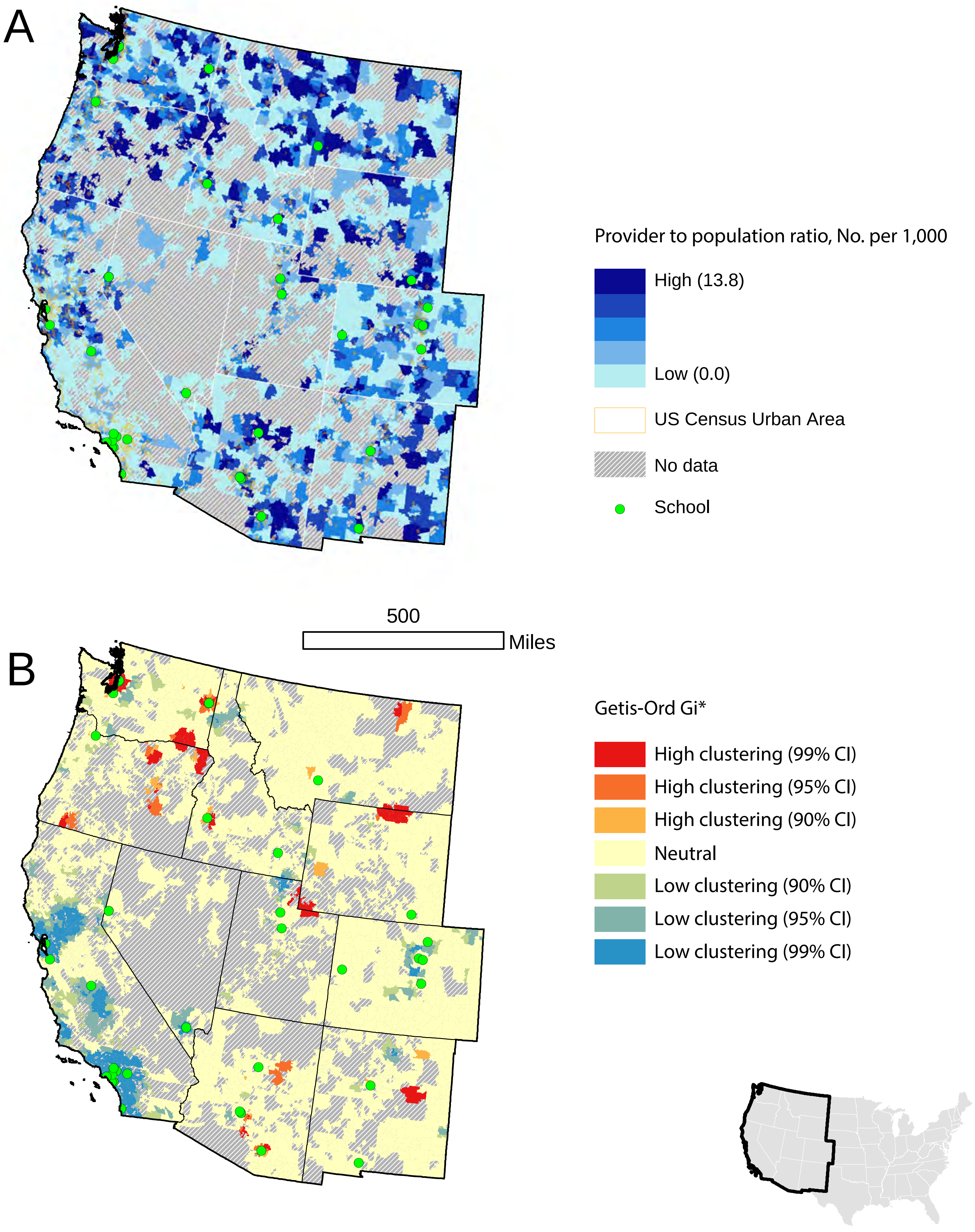

Figure 4.4. Provider to population ratio for nurse practitioners (A) and Getis-Ord Gi* statistic (B) in the West.
