# Supplementary material for: Geographic variation in spatial accessibility of U.S. healthcare providers
Source: PLoS One. 2019 Apr 9;14(4):e0215016. doi: 10.1371/journal.pone.0215016 (PMC6456202; doi:10.1371/journal.pone.0215016)
Supplement: S5 Fig — (PDF) [file pone.0215016.s005.pdf]

Chiropractor accessibility and Getis-Ord Gi* statistic by U.S. census region

Fig 5.1. Spatial accessibility for chiropractors (A) and Getis-Ord Gi* statistic (B) in the Northeast.

Fig 5.2. Spatial accessibility for chiropractors (A) and Getis-Ord Gi* statistic (B) in the Midwest.

Fig 5.3. Spatial accessibility for chiropractors (A) and Getis-Ord Gi* statistic (B) in the South.

Fig 5.4. Spatial accessibility for chiropractors (A) and Getis-Ord Gi* statistic (B) in the West.

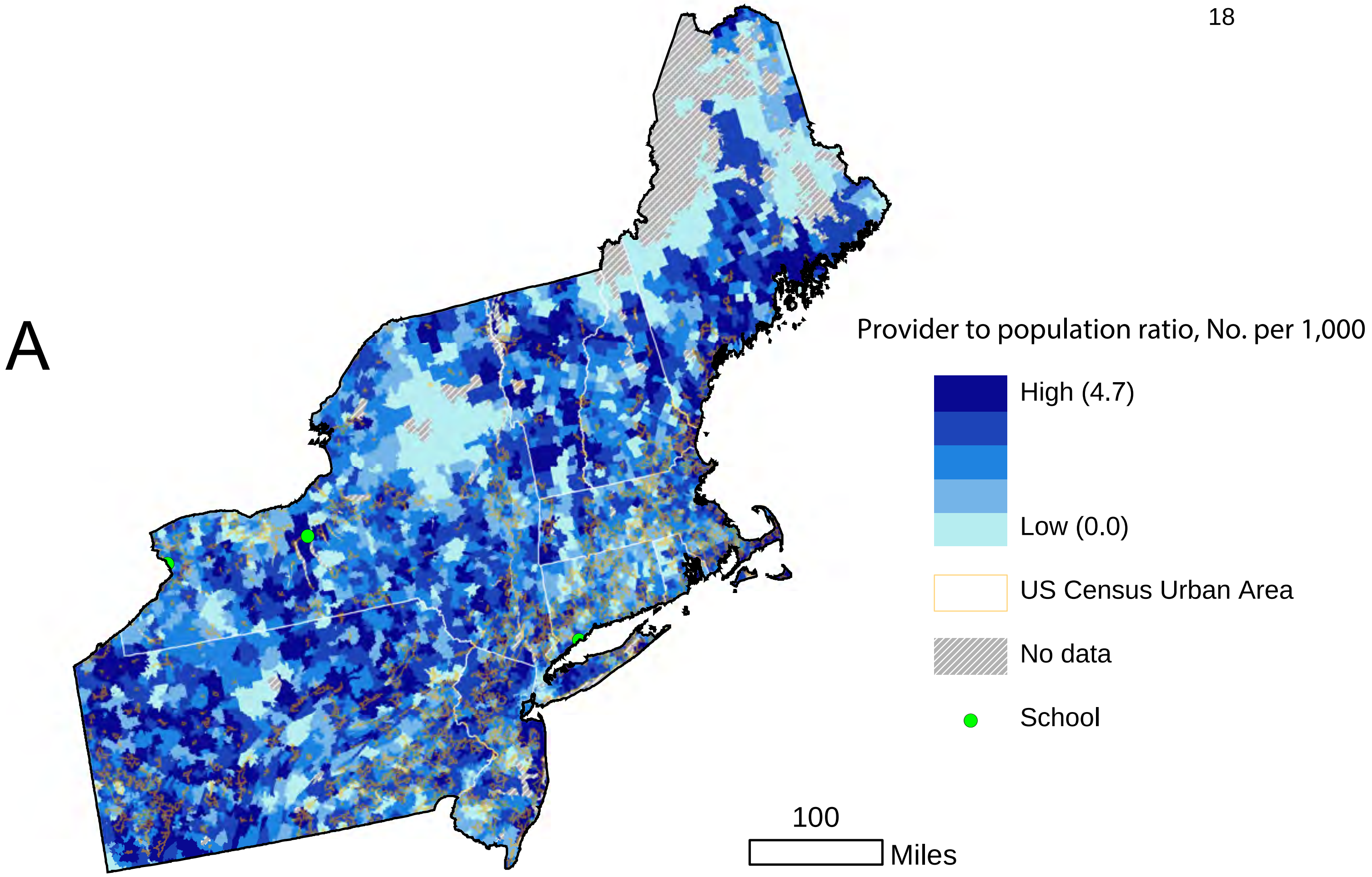

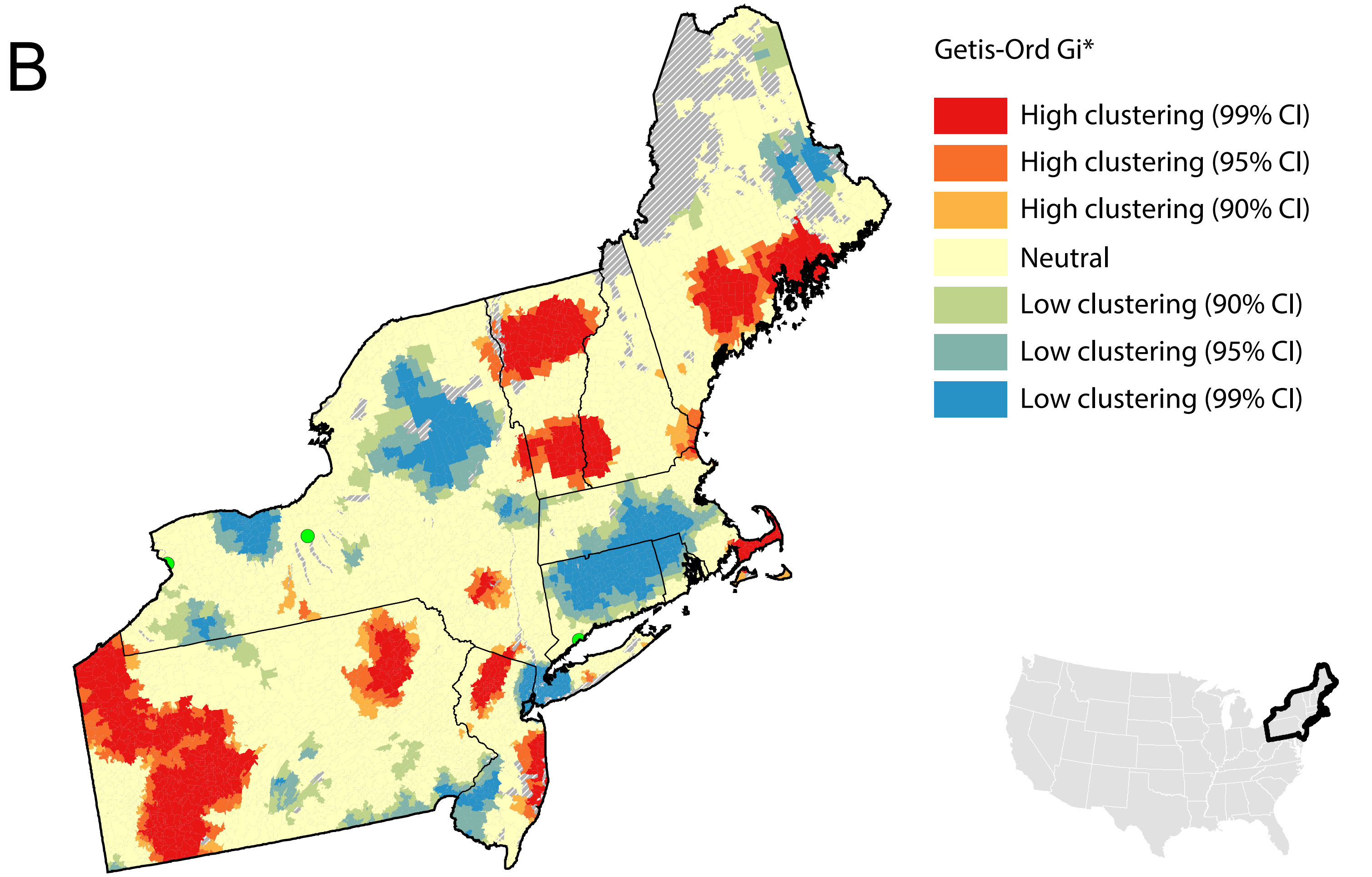

Figure 5.1. Provider to population ratio for chiropractors (A) and Getis-Ord Gi* (B) in the Northeast.

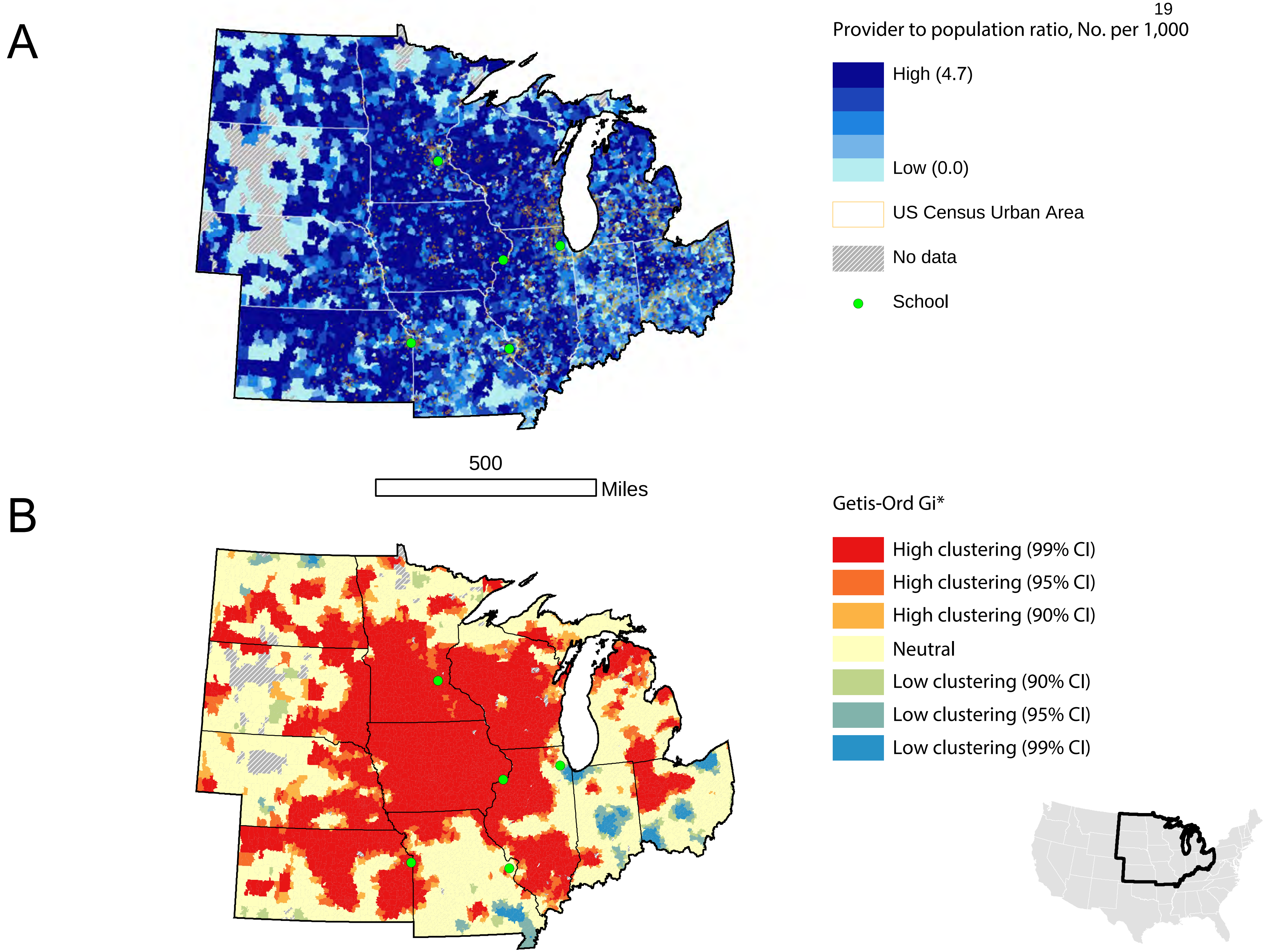

Figure 5.2. Provider to population ratio for chiropractors (A) and Getis-Ord Gi* statistic (B) in the Midwest.

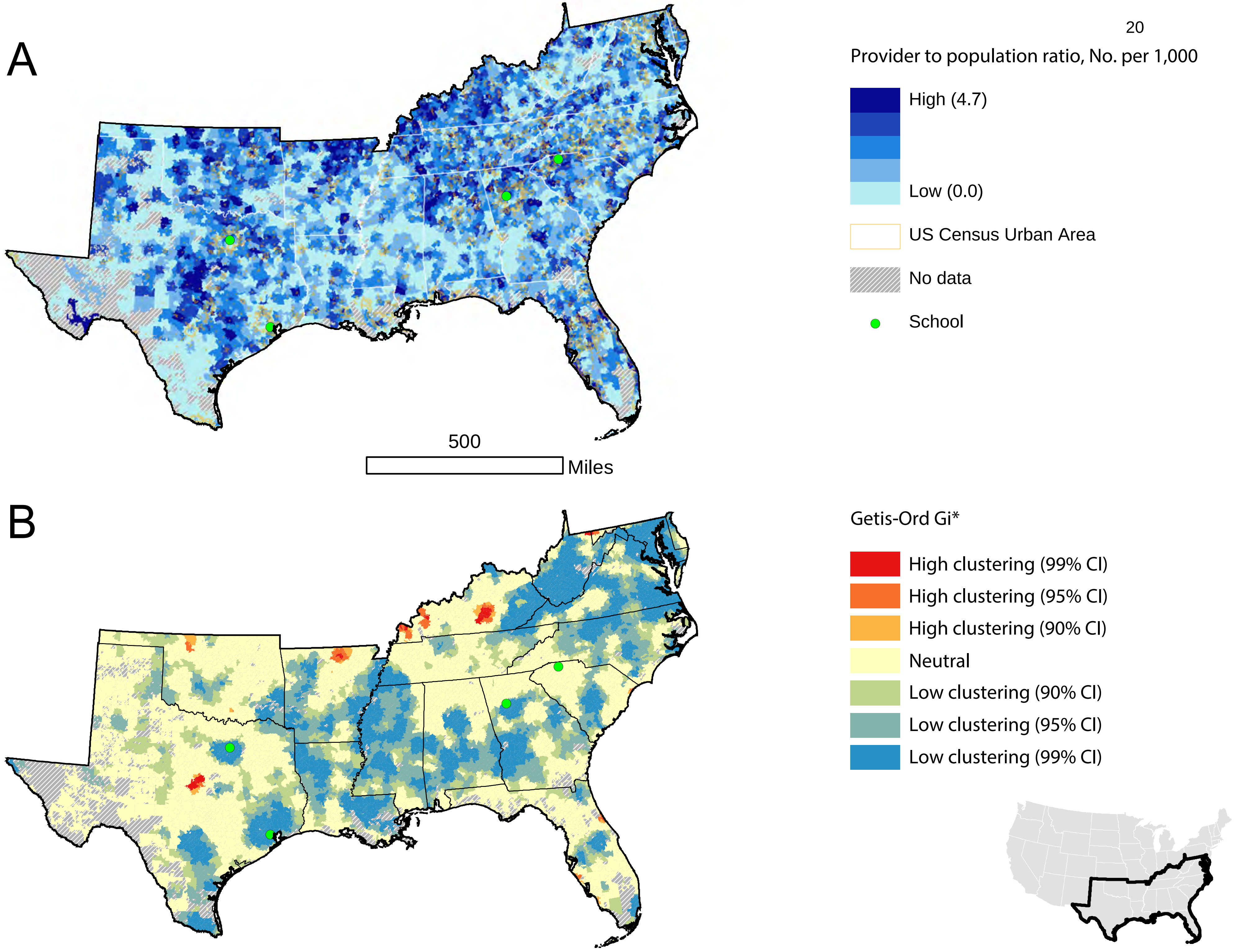

Figure 5.3. Provider to population ratio for chiropractors (A) and Getis-Ord Gi* statistic (B) in the South.

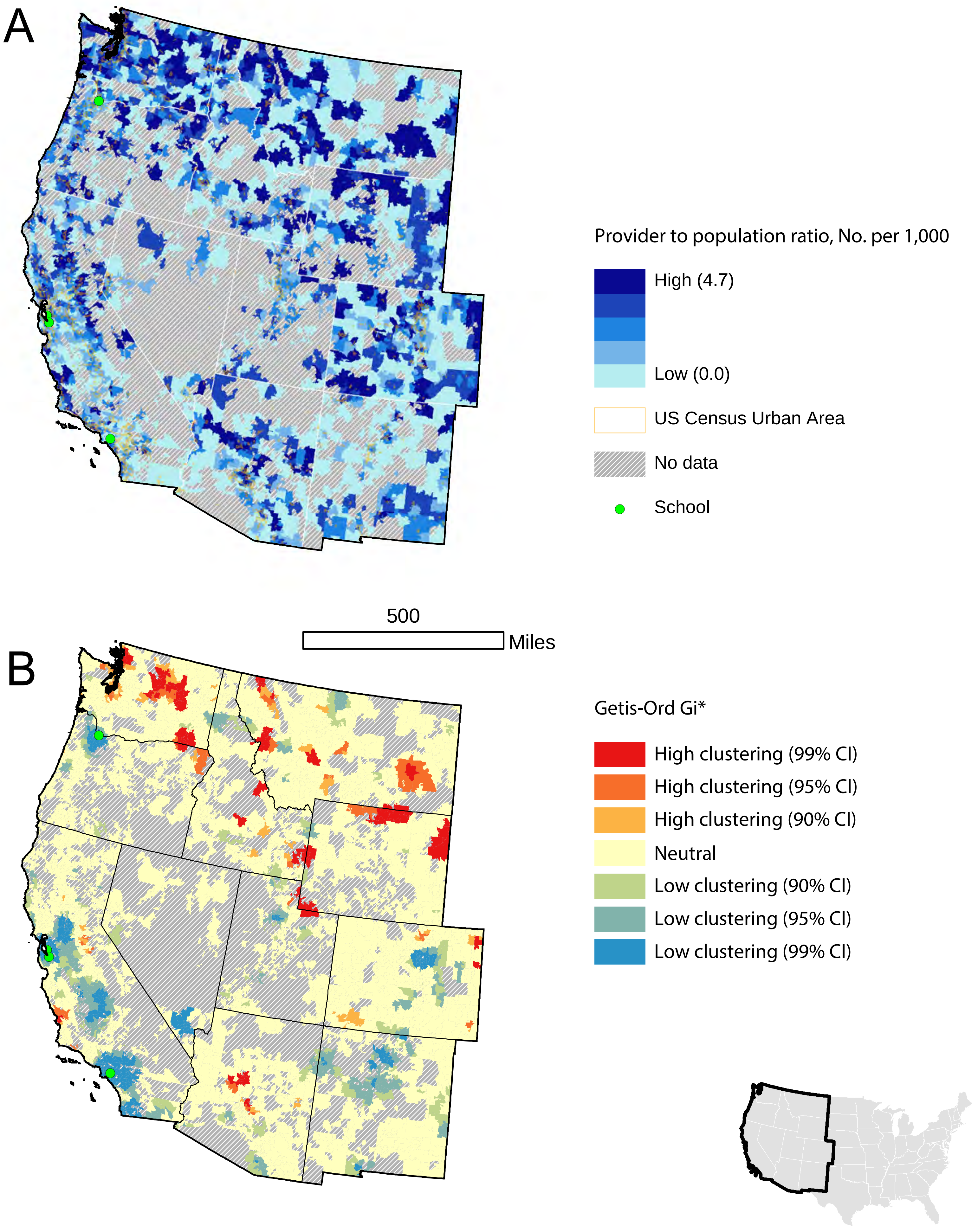

Figure 5.4. Provider to population ratio for chiropractors (A) and Getis-Ord Gi* statistic (B) in the West.
